# Sorafenib-associated Trichodysplasia Spinulosa versus Follicular Hyperkeratosis: a case report

**DOI:** 10.1093/omcr/omaf273

**Published:** 2025-12-26

**Authors:** Reem Hasan, Nemat Alsaghir, Kinda Alshawa, Sajeda Alnabelsi, Hanaa Almsokar

**Affiliations:** Department of Dermatology and Venereology, Damascus University, Damascus, Syria; Department of Dermatology and Venereology, Damascus University, Damascus, Syria; Department of Dermatology and Venereology, Damascus University, Damascus, Syria; Department of Dermatology and Venereology, Damascus University, Damascus, Syria; Department of Dermatology and Venereology, Damascus University, Damascus, Syria

**Keywords:** sorafenib, trichodysplasia spinulosa, follicular hyperkeratosis

## Abstract

Sorafenib is a multi-target kinase inhibitor used to treat advanced cancers, such as hepatocellular carcinoma. It has been associated with various cutaneous adverse events and here we report a new potential skin reaction—Trichodysplasia Spinulosa (TS)—and explore its differential diagnosis with Follicular Hyperkeratosis (FH), both possibly induced by Sorafenib. we highlight the role of sorafenib in disrupting skin keratinization process and propose a mechanism for this reaction, especially in immunosuppressed individuals.

## Introduction

The pathophysiology of sorafenib remains unclear and needs further prospective studies to clarify, but here we focus on its role as an inhibitor of angiogenesis, particularly its activity on multiple targets such as Vascular Endothelial Growth Factor Receptors 2 and 3 (VEGFR 2,3). Moreover, Vascular Endothelial Growth Factor (VEGF) is known to increase the keratinization process in the skin. By inhibiting the VEGFR, sorafenib may play a role in disrupting normal keratinization, although the mechanism remains uncertain, and that may give a comprehensive clue in sorafenib rule in *Trichodysplasia spinulosa* (TS) and Follicular Hyperkeratosis FH [1]. sorafenib has also an immunosuppression role at higher doses which explains its role in TS [2].

TS manifests as a skin eruption consisting of asymptomatic folliculocentric papules with keratotic spines, mainly on the face and neck, and less frequently on the trunk and extremities [3]. Histopathologic findings include dilated and dysmorphic hair follicles and the proliferation of inner root sheath cells with enlarged trichohyalin granules [4]. There is no specific treatment for this disorder, but topical cidofovir 3% and oral valganciclovir, in addition to reducing the cause of immunosuppression, seems beneficial [5]. On the other hand, Follicular Hyperkeratosis is another rare and benign dermatologic disorder, first recognized as a side effect of cyclosporine. Subsequent case reports have shown that it can also be a side effect of Sorafenib drug [1, 6, 7]. Clinically, it manifests as white follicular hyperkeratosis spicules, typically appearing 9 to 164 days after starting treatment [1]. Histologically it showed hyperkeratotic follicular plug and perifollicular lymphocytic infiltrate [1, 7]. Treatment also depends on discontinuing the drug plus keratolytic agents.

### Case presentation

**Figure 1:**
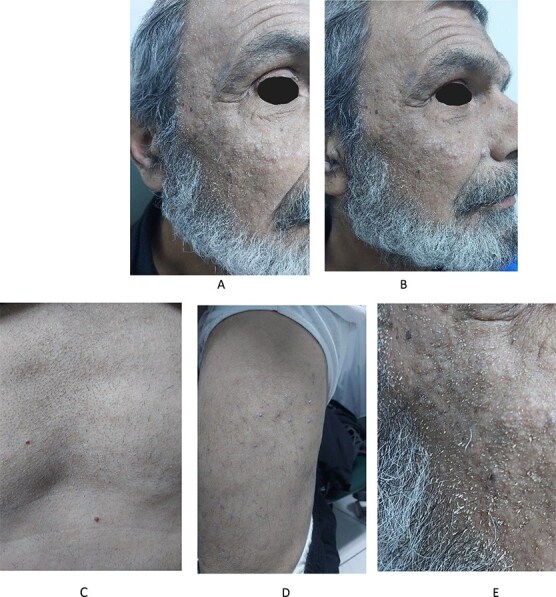
Papules with folliculocentric spikes A, B) on the face. C) on the chest. D) on the extremities. E) Close-up view.

**Figure 2:**
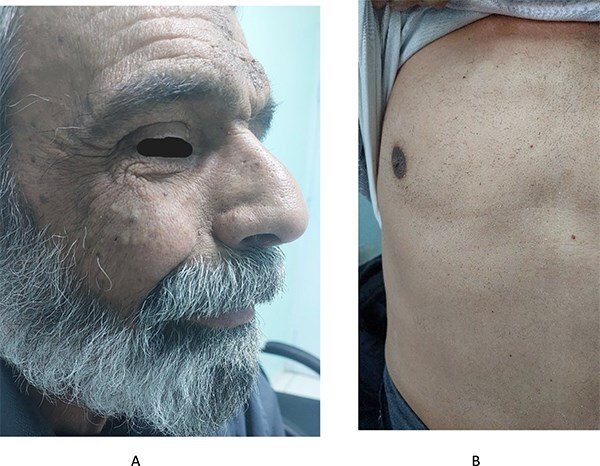
Improvement after treatment. A)- on the face. B)- on the chest.

**Figure 3:**
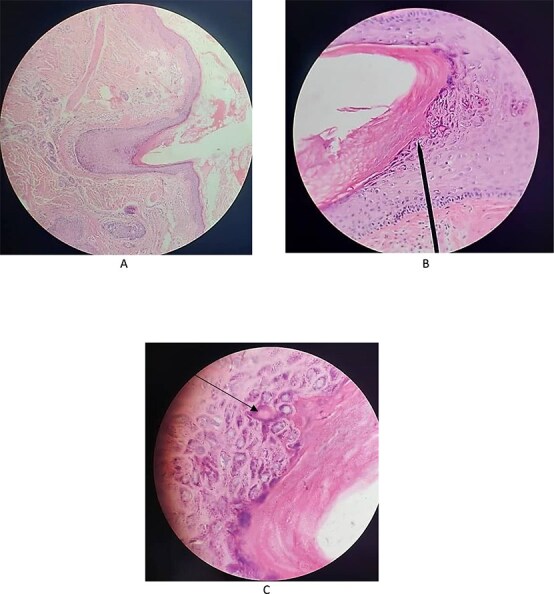
A, B)- dilated hair follicles, keratin plugging, and numerous large eosinophilic trichohyalin granules within the inner root sheath cells. C)- inclusion bodies.

A 65-year-old male smoker presented to the Hospital of Dermatology in Damascus with a skin eruption involving the face, neck, and trunk. As documented in the patient’s medical history, he was diagnosed previously with liver cirrhosis, which had progressed to hepatocellular carcinoma. In light of these findings, the patient was treated with sorafenib. However, after one week of treatment, he developed a skin rash, which led his gastroenterologist to ask for a dermatology consultation besides discontinuing the previously mentioned medication. On physical examination, the patient exhibited multiple follicular papules with a central keratin spine on various areas, including the face, neck, trunk and extremities (Fig. 1). The differential diagnoses were made Sorafenib-associated Follicular Hyperkeratosis and Trichodysplasia Spinulosa. Skin biopsy was achieved and it was more compatible with Trichodysplasia Spinulosa. The histological findings showed dilated hair follicles, keratin plugging, and numerous large eosinophilic trichohyaline granules within the inner root sheath cells in addition to inclusion bodies in some sections (Fig. 3). Although the patient previously discontinued his drug based on his gastroenterologist’s decision for nearly a month without improvement, our treatment plan in addition to discontinuing the accused drug, was oral acyclovir (800 mg five times per day for one week). Remarkable improvement was observed After one week of treatment, suggesting but not confirming a viral contribution (Fig. 2). The final diagnosis and decided treatment were made based on clinical and histological findings at first, and with improvement, after treatment was acyclovir, the diagnosis is more likely Trichodysplasia Spinulosa.

## Discussion

This case illustrates a rare cutaneous adverse event likely induced by sorafenib, Sorafenib is a well-established oral multi-kinase inhibitor approved for unresectable hepatocellular carcinoma. While commonly associated with hand-foot skin reactions, rash, and alopecia [8], other dermatologic adverse effects such as FH have been documented [1].

In this case the patient developed keratotic follicular papules shortly after starting sorafenib. Given the overlapping features of TS and FH, a skin biopsy was essential for diagnosis. As outlined in Table 1, both conditions can present with spiny follicular lesions, and both have been reported in association with immunosuppressants and sorafenib. However, the presence of enlarged trichohyalin granules and inclusion bodies is characteristic of TS.

**Table 1:** Comparative features of Trichodysplasia Spinulosa and Follicular Hyperkeratosis.

	Trichodysplasia Spinulosa	Follicular Hyperkeratosis
Clinical manifestations	painless erythematous papules on the face with folliculocentric keratin spines.	Painless flesh-colored or white, follicular hyperkeratotic spicules.
Histopathological manifestations	dilated hair follicles, keratin plugging, abundant dystrophic and cornfield inner root sheath epithelium, and numerous, large eosinophilic trichohyalin granules within the inner root sheath cells.	Dilated hair follicles with hyperkeratotic follicular plug and perifollicular lymphocytic infiltrate.
Associated conditions	Immunosuppressive patients such as: solid organ transplant, medications (cyclosporine, vismogedib), hematolymphoid malignancy, and HIV infection.	Related to drug (sorafenib, vemurafenib, glasdegib, cyclosporine, acitretin) and non-drug conditions (Paraproteinemia, multiple myeloma, Crohn’s disease, hypovitaminosis A, chronic renal failure, human immunodeficiency virus infection, cryoglobulinemia, Sezary syndrome and lymphoma).
Treatment	Discontinue the accused medication plus anti-viral medications.	Discontinue the medication plus keratolytic agents.
Locations	The central face is most frequently affected, but other areas such as the ears, trunk, and extremities may also be involved.	involving the face, scalp, upper trunk, and upper arms.

Although a PCR test for TSPyV would be ideal to confirm TS, this was not available. Nevertheless, the clinical presentation, histopathological features, and rapid response to antiviral therapy strongly support a diagnosis and diagnostic rationale are summarized in the comparative table (Table 1), which was pivotal in reaching our final conclusion.

Our findings are consistent with prior studies that describe sorafenib-associated spiny follicular eruptions. Frank et al. reported FH-like reactions in nine patients on sorafenib, resolving upon discontinuation and recurring with rechallenge [1]. However, our case differs by showing histological features more specific to TS. Interestingly, there are overlapping reports in literature suggesting a spectrum between TS and FH, possibly representing a continuum of the same disease under different levels of immunosuppression.

Limitations of this report include the absence of PCR testing to confirm TSPyV, which limits virological certainty. Nevertheless, the strength of histological evidence, clinical correlation, and therapeutic response mitigate this limitation.

The differential diagnosis of Trichodysplasia Spinulosa includes Follicular Hyperkeratosis. Both conditions feature spiny keratotic plugs and are accompanied by tiny follicular papules, as well as nonscarring alopecia. Histologically, they both present with dilated hair follicles. However, TS is characterized by the presence of the TSPyV. in one study involving FH, viral particles were found in a patient with lymphoma [9]. additionally, the distribution of lesions in FH is typically on the mid face and it features larger red to white papules and nodules. Interestingly, there exists a case report about follicular hyperkeratosis associated with sorafenib treatment. Our case also presented Trichodysplasia Spinulosa in a patient undergoing the same medication. While the clinical and histological features of both conditions are quite similar, plus both of these conditions have been identified as side effects of cyclosporin treatment. Therefore, we propose that these two entities may represent the same disease manifesting in different severities.

## Conclusions

This case suggests that sorafenib may induce TS in addition to FH. We propose that TS and FH may represent a spectrum of the same pathological process modulated by the degree of immunosuppression and viral activation. Future studies, including PCR confirmation and broader case series, are needed to substantiate this hypothesis. A similar perspective was suggested by Kazem et al., emphasizing the role of TSPyV in TS pathology [10].
